# Ectopic adrenocortical adenoma in the renal hilum: a case report and literature review

**DOI:** 10.1186/s13000-016-0490-6

**Published:** 2016-04-19

**Authors:** Yang Liu, Yue-Feng Jiang, Ye-Lin Wang, Hong-Yi Cao, Liang Wang, Hong-Tao Xu, Qing-Chang Li, Xue-shan Qiu, En-Hua Wang

**Affiliations:** Department of Pathology, The First Affiliated Hospital and College of Basic Medical Sciences, China Medical University, Shenyang, 110001 China; Institute of Pathology and Pathophysiology, China Medical University, Shenyang, 110001 China

**Keywords:** Ectopic adrenocortical adenoma, Ectopic adrenal gland, Ectopic oncolytic adrenocortical neoplasm, Renal hilum, Oncocytes

## Abstract

**Background:**

Ectopic (accessory) adrenocortical tissue, also known as adrenal rests, is a developmental abnormality of the adrenal gland. The most common ectopic site is in close proximity to the adrenal glands and along the path of descent or migration of the gonads because of the close spatial relationship between the adrenocortical primordium and gonadal blastema during embryogenesis. Ectopic rests may undergo marked hyperplasia, and occasionally induce ectopic adrenocortical adenomas or carcinomas.

**Case presentation:**

A 27-year-old Chinese female patient who presented with amenorrhea of 3 months duration underwent computed tomography urography after ultrasound revealed a solitary mass in the left renal hilum. Histologically, the prominent eosinophilic tumor cells formed an alveolar- or acinar-like configuration. The immunohistochemical profile (alpha-inhibin+, Melan-A+, synaptophysin+) indicated the adrenocortical origin of the tumor, diagnosed as ectopic adrenocortical adenoma. The patient was alive with no tumor recurrence or metastasis at the 3-month follow-up examination.

**Conclusions:**

The unusual histological appearance of ectopic adrenocortical adenoma may result in its misdiagnosis as oncocytoma or clear cell renal cell carcinoma, especially if the specimen is limited. This case provides a reminder to pathologists to be aware of atypical cases of this benign tumor. Although uncommon, an ectopic adrenal lesion should be included in the differential diagnosis of tumors involving the renal hilum. A misdiagnosis of this benign condition as a malignant renal tumor may have severe consequences for the patient, including unnecessary radical nephrectomy. Preoperative biopsy and appropriate immunohistochemical staining will assist in determining the origin and nature of the tumor and in avoiding intraoperative uncertainty.

## Background

Ectopic adrenal rests have been reported from a variety of anatomic sites, including the celiac plexus, kidney, testis, epididymis, broad ligament, the canal of Nuck, hernial and hydrocele sacs, the mesoappendix, liver, lung, intradural space and brain [1–7]. Cortical tissue seems to be the sole component of ectopic adrenal rests, as there have been no reports of accompanying medullary tissue. Occasionally, ectopic rests undergo marked hyperplasia and develop into ectopic adrenocortical adenomas and carcinomas [7]. Because the clinical features of ectopic adrenocortical neoplasms depend on their hormone secretion status, these tumors may be functional or non-functional. However, in contrast to their functional counterparts, non-functional ectopic adrenocortical neoplasms may go undetected because these patients are mostly asymptomatic. Here, we report a case of ectopic adrenocortical adenoma located in the renal hilum, a rare site for the occurrence of this tumor and one that may have easily led to its misdiagnosis as renal cancer and therefore to radical nephrectomy. This case provides a reminder to pathologists to be aware of ectopic adrenocortical adenoma, as these patients can be treated with nephron-sparing surgery. Preoperative biopsy will aid in the correct diagnosis.

## Case presentation

### Clinical history

**Fig. 1:**
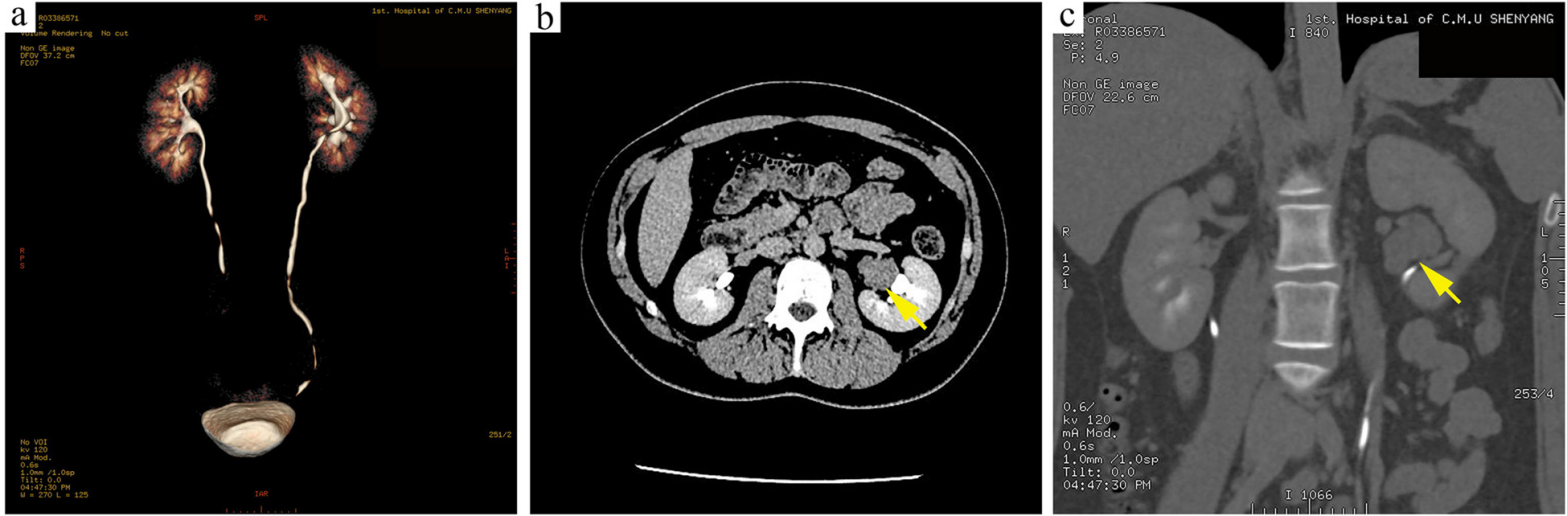
Imaging examination results of the patient. **a** Computed tomography (CT) urography and three dimensional reconstruction show the ureters bilaterally, without any obvious expansion. Enhanced CT and coronal reconstruction showing **b** a soft-tissue mass with a maximum diameter of 2.7 cm in the left renal hilum, near the renal pelvis and **c** an atrophic bilateral adrenals in sagittal view

A 27-year-old Chinese female complained of amenorrhea of 3 months duration. Her medical history and that of her family was unremarkable. Her body mass index (BMI) and blood pressure (BP) were within the normal range (BMI: 19, height 165 cm, body weight: 62 kg, BP: 120/85 mmHg). Virilization and other clinical hormonal abnormalities were not noted, except for a borderline elevation of testosterone. Multislice spiral computed tomography (CT) urography revealed a well-circumscribed, round, soft-tissue mass with a maximum diameter of 2.7 cm in the left renal hilum, near the renal pelvis and atrophic bilateral adrenals. The CT value for the unenhanced mass was 35 HU; following contrast agent administration, it increased to 161 HU. Multislice spiral CT urography showed no abnormalities in kidney blood flow or renal perfusion. Renal function was normal, except for a slight delay in renal excretion (Fig. 1). The tumor was clinically diagnosed as an angiomyolipoma and was excised in urinary surgery. During the operation, the mass in the left renal hilum was fully resected, sparing the kidney and was sent for pathology examination. Rapid intraoperative pathological diagnosis suggested oncocytoma, but paraganglioma and renal cell carcinoma could not be excluded. Based on the postoperative pathology results, the diagnosis was ectopic adrenocortical adenoma. The patient is alive with no tumor recurrence or metastasis after 3 months of follow-up.

### Materials and methods

The tumor tissues were fixed in 10 % formalin and embedded in paraffin. Four-micrometer sections were cut from each paraffin block. One section was stained with hematoxylin-eosin (H&E); the others were stained for immunohistochemistry using the streptavidin-peroxidase system (Ultrasensitive; Mai Xin Inc., Fuzhou, China) according to the manufacturer’s instruction. Commercially available, prediluted monoclonal antibodies against the following antigens were used to evaluate the specimen: pan-cytokeratin (AE1/AE3), vimentin, synaptophysin, CD56, neuron specific enolase (NSE), alpha-inhibin, Melan-A, CD34, S100, PAX8, chromogranin A, Ki-67 and anti-mitochondrial antibody (AMA; Millipore, Darmstadt, Germany). For the negative controls, the primary antibody was replaced with PBS.

### Gross features

Grossly, the mass was solid, round and well circumscribed; its cut surface was yellow and brown. The mass measured 2.5 cm at its largest diameter and weighed 8 g. Neither necrosis nor hemorrhage was identified.

### Microscopic features

**Fig. 2:**
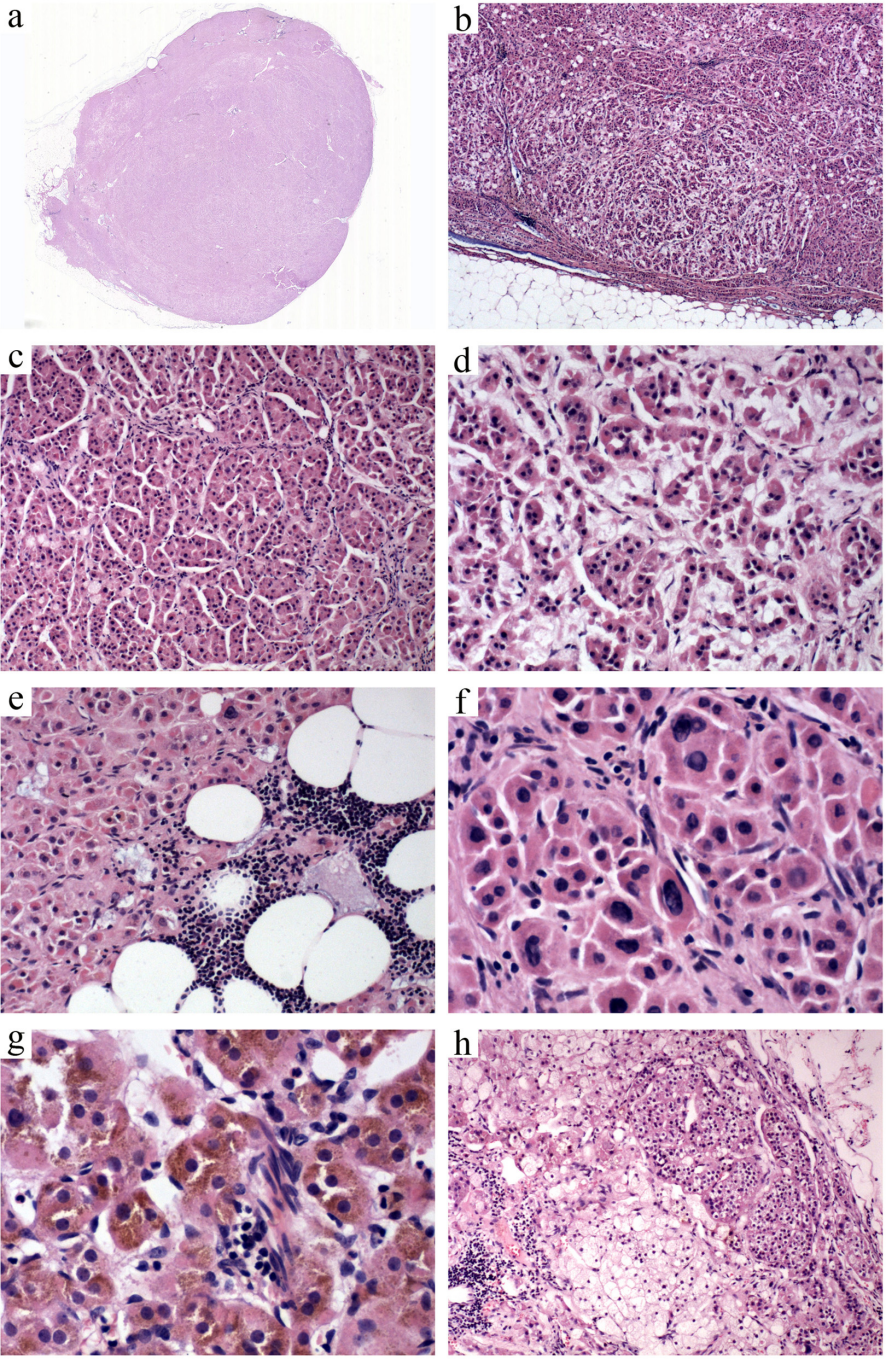
Histological features. The tumor is circumscribed by a discontinuous capsule (**a**) and is clearly demarcated from the surrounding adipose tissue (**b**). **c** The tumor cells are mainly arranged in nests with an alveolar- or acinar-like configuration. These acini are surrounded by an open vascular network. **d** Foci of edematous stroma are seen within the tumor. **e** The focal infiltration of mature lymphocytes is especially prominent in the areas undergoing lipomatous or myelolipomatous metaplasia. **f** Bizarre nuclear forms are occasionally present, especially in the area of diffuse oncocytes. The cells contain eosinophilic nuclear pseudoinclusion bodies. **g** Lipofuscin pigmentation is detected in focal areas. **h** The peripheral adipose tissue contains foci of ectopic adrenal rests

Histologically, the tumor was well circumscribed and composed of cells mainly arranged in nests forming an alveolar- or acinar-like configuration (Fig. 2a–c) and surrounded by an open vascular network. Foci of edematous stroma within the tumor (Fig. 2d) and oncocytes with a diffuse growth pattern along its periphery were observed. Focal infiltrations of mature lymphocytes were especially prominent in areas undergoing lipomatous or myelolipomatous metaplasia (Fig. 2e). The tumor was mainly composed of cells with an abundant eosinophilic cytoplasm. Focal areas of pale-staining clear cells with a lipid-filled cytoplasm were identified but were rare. Bizarre nuclear forms were occasionally seen in areas with diffuse tumor cells (Fig. 2f). Eosinophilic nuclear pseudoinclusion bodies and lipofuscin pigmentation were also detected (Fig. 2g). However, mitoses were exceptionally rare or absent. The peripheral adipose tissue contained foci of ectopic adrenal rests (Fig. 2h).

### Immunohistochemistry

**Fig. 3:**
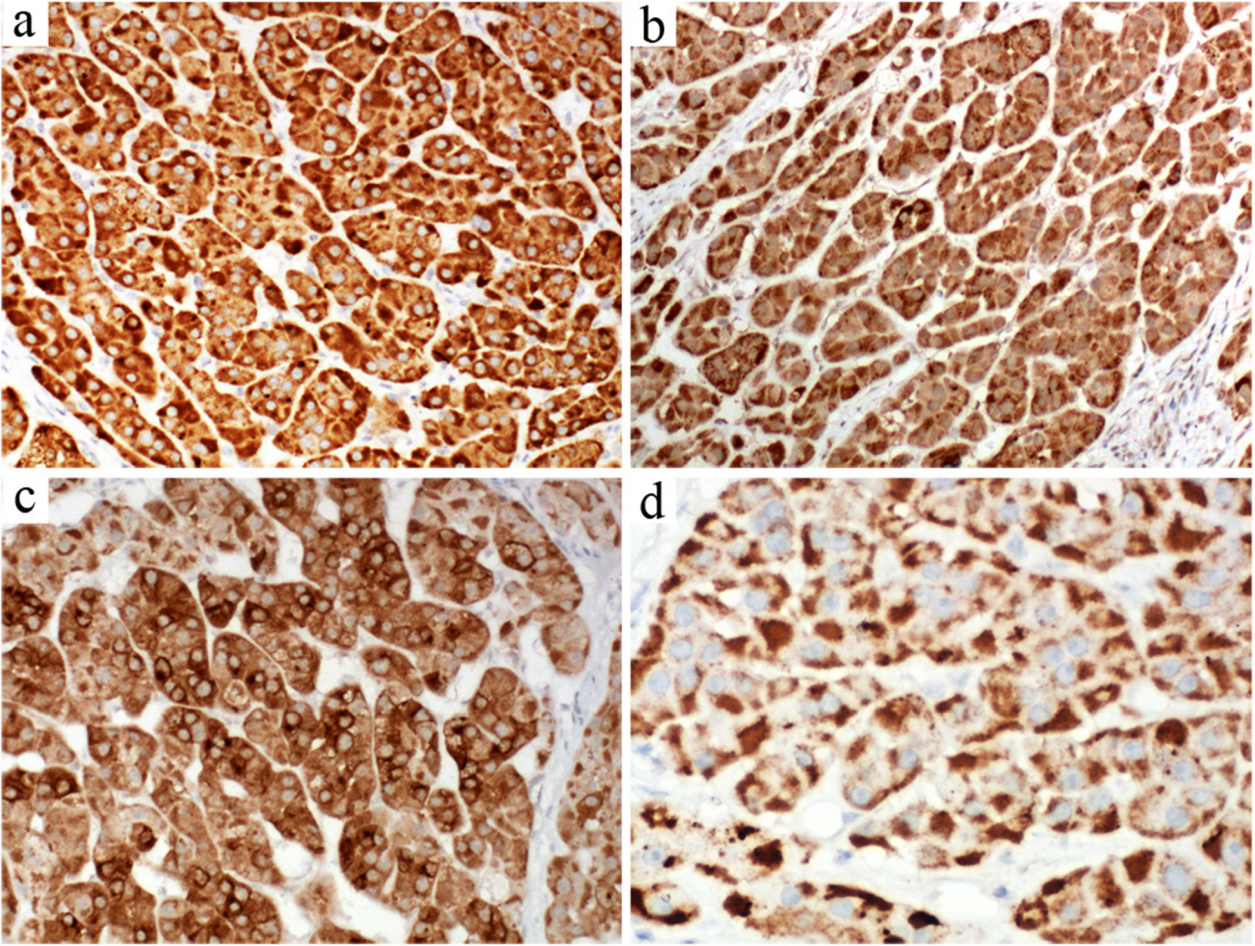
Immunohistochemical staining. **a** The tumor cells are markedly and diffusely positive for alpha-inhibin. Their cytoplasm shows positive staining for **b** Melan-A and **c** synaptophysin. **d** Diffuse fine granular reactivity with an anti-mitochondrial antibody is seen in eosinophilic cells

The tumor cells were markedly and diffusely positive for vimentin, alpha-inhibin (Fig. 3a), Melan-A (Fig. 3b), synaptophysin (Fig. 3c), NSE and CD56, and focally positive for pan-cytokeratin (AE1/AE3), but negative for PAX8, S100 and chromogranin A. The sustentacular pattern of S100 staining, typical in paraganglioma, was not observed in this case. Rather, the immunohistochemical profile was consistent with an adrenocortical origin of this tumor. In addition, a fine granular AMA immunoreactivity was detected in diffusely arranged eosinophilic cells located in peripheral regions of the tumor (Fig. 3d). The Ki-67 labeling index was < 1 %.

## Discussion

Adrenocortical primordium (seen as a thickening of the coelomic mesothelium) is formed by the invagination of the coelomic epithelium, adjacent to the region where the gonadal blastema arises, on approximately day 30 of gestation. During gonadal migration, fragments of adrenocortical tissue may be scattered along the descending path and form ectopic adrenal glands [5, 8]. Although most of these fragments settle in the vicinity of the adrenal gland or along its migratory course, in rare cases, ectopic adrenal tissue is found at distant sites, such as the lung and even the intradural space and brain [1–7]. Ectopic adrenocortical neoplasms can arise from these ectopic adrenal rests, albeit very rarely [7]. Our Discussion includes a literature review of the rare cases of ectopic adrenocortical adenoma involving the renal hilum. The reported cases [5, 9, 10] published in the English-language literature are summarized in Table 1.

**Table 1:** Reported cases of ectopic adrenocortical adenoma in the renal hilum in English-language literatures and their prognosis

Case	Authors	Age	Sex	Location	Largest diameter of the tumor (cm)	Treatment	Follow-up
1	Ayala et al. 2000 [10]	63	F	Left renal hilum	3.5	Tumor resection with intraoperative endoscopy sparing the kidney	NED, 9 months
2	Wang et al. 2012 [9]	38	M	Anterior of left renal hilum	5.3	Tumor resection sparing the kidney	ND
3	Tong et al. 2014 [5]	53	F	Left renal hilum	3.5	Tumor resection sparing the kidney	Tumor recurrence 2 years after the first surgery in 2010
4	Current case	27	F	Left renal hilum	2.5	Tumor resection sparing the kidney	NED, 3 months

*M* male, *F* female, *NED* no evidence of disease, *ND* not described

Tumors of the renal pelvis are limited in their histological type, especially those in the soft tissue near the hilum. Urothelial and renal neoplasms should be considered first, whereas other rare neoplasms include pelvic lipomatosis or fibrolipomatosis, urinoma, angiomyolipoma, nephrogenic adenoma, solitary fibrous tumor, leiomyoma, neurofibroma and hemangioma [11]. In our patient, the tumor was well circumscribed and composed of cells with a prominent eosinophilic cytoplasm and forming an alveolar- or acinar-like configuration. Combined with the characteristics described above and the focal areas of edematous stroma, oncocytoma was the most likely diagnosis. The differential diagnosis included paraganglioma and renal cell carcinoma. Slides prepared from frozen sections favored oncocytoma but did not exclude paraganglioma and renal cell carcinoma. The whole mass was sampled and H&E-stained sections were prepared postoperatively. An examination of the slides led to the identification in one of them of ectopic adrenal rests within the peripheral adipose tissue, adjacent to the tumor. This finding suggested that the tumor was an ectopic adrenocortical neoplasm arising from ectopic adrenocortical rests. We therefore examined the tumor tissue using a panel of immunohistochemical markers. The immunophenotype (AE1/AE3-, vimentin+, PAX8-, alpha-inhibin+, Melan-A+, synaptophysin+ and NSE+) did not support a renal origin of the tumor. In addition, neither CT nor CT urography revealed a primary mass in the renal parenchyma, and both renal function and renal perfusion were normal. Because these results seemed to rule out a renal neoplasm, we excluded the diagnosis of renal oncocytoma and renal cell carcinoma. The immunophenotype (chromogranin A- and S100-) and the absence of hypertension in the patient’s history ruled out a diagnosis of oncocytic paraganglioma. The final immunoprofile instead supported the adrenocortical origin of the tumor.

Adrenocortical adenoma must be distinguished from adrenocortical adenocarcinoma once an adrenocortical origin has been confirmed. Most adrenocortical adenocarcinomas are larger than adenomas, but they may overlap in size. Adrenocortical adenomas lack the histological features of malignancy, such as vascular invasion, necrosis, fibrous bands, capsular invasion, an increased mitotic rate, atypical mitoses and nuclear atypia. Nonetheless, distinguishing these benign tumors from malignant ones is difficult in some cases [1, 7, 11]. The Weiss histopathological criteria [12] are commonly used to assess the malignancy of adrenocortical tumors. In 2002, the criteria were modified and improved by Aubert [13]. The original and modified Weiss criteria [12, 13] are summarized in Table 2. In the tumor removed from our patient, there was no evidence of necrosis or hemorrhage on either micro- or macroscopic examination, nor were fibrous bands or capsular invasion observed. Although occasional bizarre nuclear forms were detected, mitoses were exceptionally rare or absent, and the Ki-67 index was < 1 %. These histological features favored a diagnosis of adenoma rather than adenocarcinoma. Foci of endocrine atypia are not uncommon in benign endocrine lesions, although nuclear pleomorphism and eosinophilic nuclear pseudoinclusions are frequent findings in oncocytic adrenocortical neoplasm. Only monotonous sheets of cells with a high nuclear-to-cytoplasmic ratio should raise strong suspicion of malignancy, whereas endocrine atypia is considered characteristic of benign endocrine lesions [1, 14–16]. For these reasons, the diagnosis in the present case was adenoma rather than adenocarcinoma. Although adrenal cortical adenomas usually consist of sheet-like, clear to eosinophilic, compact tumor cells, the cells in our patient’s tumor were arranged in nests with an alveolar or acinar configuration and surrounded by an open vascular network rather than a delicate capillary network. Foci of edematous stroma were also observed. These uncommon findings were initially confusing. Erickson [1] described the variable cytoplasmic clearing of tumor cells and the intermingling of cells with a more or less distinct morphology. In addition, myxoid adrenocortical tumors, while rare, can occur.

**Table 2:** Original and modified Weiss criteria for evaluating malignancy in adrenal cortical neoplasms

Original Weiss criteria	Modified Weiss criteria
Nuclear grade III or IV based on Fuhrman criteria	Mitotic rate >5 per 50 high-power fields
>5 mitotic figures/50 HPF (40× objective), counting 10 random fields in area of greatest number of mitotic figures on 5 slides with greatest number of mitoses	Cytoplasm (clear cells comprising 25 % or less of the tumor)
Presence of atypical mitotic figures (abnormal distribution of chromosomes or excessive number of mitotic spindles)	Abnormal mitoses
Clear or vacuolated cells comprising 25 % or less of tumor	Necrosis
Diffuse architecture (more than 1/3 of tumor forms patternless sheets of cells; trabecular, cord, columnar, alveolar or nesting pattern is not considered to be diffuse)	Capsular invasion
Microscopic necrosis	
Venous invasion (veins must have smooth muscle in wall; tumor cell clusters or sheets forming polypoid projections into vessel lumen or polypoid tumor thrombi covered by endothelial layer)	
Sinusoidal invasion (sinusoid is endothelial lined vessel in adrenal gland with little supportive tissue; consider only sinusoids within tumor)	
Capsular invasion (nests or cords of tumor extending into or through capsule with a stromal reaction); either incomplete or complete	
Calculate: score of 3 or more suggests malignancy	Calculate: 2× mitotic rate criterion + 2× clear cytoplasm criterion + abnormal mitoses + necrosis + capsular invasion (score of 3 or more suggests malignancy)
Each criterion is scored 0 when absent and 1 when present in the tumor	Each criterion is scored 0 when absent and 1 when present in the tumor

The final problem was to distinguish this tumor from oncocytic adrenocortical neoplasm and adrenocortical oncocytoma. The neoplastic cells of oncocytoma are exclusively oncocytes, with an abundant, eosinophilic and granular cytoplasm. The cells are predominantly arranged in a diffuse or solid pattern, although foci with an alveolar configuration have been described [14–17]. Oncocytic adrenocortical neoplasm may in fact be a morphological variant of adrenocortical neoplasm, regardless of its functionality, while adrenocortical oncocytoma is a purely non-functioning adrenocortical neoplasm. However, this distinction remains a matter of debate. Wong et al. [16] reported 13 cases of oncocytic adrenocortical neoplasm; seven of the respective patients had either clinical symptoms or high levels of hormone secretion. Therefore, oncocytic adrenocortical neoplasm may be a subtype of adrenocortical adenoma characterized by an oncocytic morphology. Although in the tumor from our patient a diffuse or solid pattern was seen in focal areas, it was not the main component. Accordingly, the more appropriate diagnosis was adrenocortical adenoma, and not oncocytic adrenocortical neoplasm.

Ectopic adrenal rests may undergo remarkable hyperplasia in patients with Nelson syndrome or in association with increased adreno-cortico-tropic-hormone production. However, ectopic adrenal cortical adenomas and carcinomas arising from ectopic rests are extremely rare. Some ectopic neoplasms can lead to Cushing’s syndrome, hyperaldosteronism or virilization or feminization, while others are nonfunctioning or asymptomatic, with only biochemical evidence of hormone hypersecretion [1, 7, 11]. Based on her medical history, our patient had no clinical symptoms suggesting Cushing’s syndrome, hyperaldosteronism or virilization, but she did have a borderline elevation of testosterone and amenorrhea of 3 months duration. The latter may have reflected hormone secretion by the ectopic adrenocortical adenoma.

## Conclusion

This case provides a reminder to pathologists to be aware of atypical ectopic adrenocortical adenoma and to include an ectopic adrenal lesion in the differential diagnosis of tumors involving the renal hilum. The failure to distinguish this benign tumor from a malignant renal tumor may have severe consequences, including unnecessary radical nephrectomy. Preoperative biopsy and appropriate immunohistochemical staining can aid in determining the origin and nature of the tumor and thereby avoid intraoperative uncertainty.

### Ethical approval and consent to participate

Ethical approval for this study was obtained from the Local Trials Committee of the China Medical University.

### Consent for publication

Informed consent was obtained from the patient for the publication of this case report and any accompanying images. A copy of the written consent is available for review by the Editor-in-Chief of this journal.

## Abbreviations

AMA: anti-mitochondrial antibody
CT: computed tomography
H&E: hematoxylin-eosin
